# Comparative Outcomes and Safety of Radiofrequency Ablation and Cryoablation for Lumbar Facet Joint Degeneration: A Single-Center Retrospective Cohort Study with 24-Month Follow-Up

**DOI:** 10.3390/jcm14207408

**Published:** 2025-10-20

**Authors:** Ahmet Yilmaz, Cagatay Kucukbingoz

**Affiliations:** Ministr of Health Adana City Training & Research Hospital, 01370 Adana, Turkey; ckbingoz.md@gmail.com

**Keywords:** lumbar facet joint, chronic low back pain, radiofrequency ablation, cryoablation, retrospective cohort, MCID, PASS

## Abstract

**Background:** Lumbar facet joint degeneration is a common source of chronic low back pain. Radiofrequency (RF) ablation is widely used, whereas cryoablation (CA) remains less extensively investigated. Evidence directly comparing the long-term outcomes of these two modalities is scarce, particularly beyond 12 months. **Methods:** This single-center retrospective cohort study analyzed patients with lumbar facet joint degeneration who underwent RF (n = 97) or CA (n = 83). Clinical data were retrieved from institutional records. Pain intensity (VAS), functional outcomes (ODI and RMDQ), and quality-of-life measures (EQ-5D and SF-36) were assessed at baseline and at 1, 3, 6, 12, 18, and 24 months. The primary outcome was change in ODI; secondary outcomes included VAS, RMDQ, quality of life, and complications. Clinically meaningful improvement was defined according to Minimal Clinically Important Difference (MCID) and Patient Acceptable Symptom State (PASS) thresholds. **Results:** Both RF and CA significantly improved pain and function compared with baseline. RF demonstrated superior reductions in ODI and VAS at 12 and 18 months (*p* < 0.05); however, by 24 months, treatment effects had diminished, and no significant differences remained (*p* > 0.05). Quality-of-life improvements plateaued after 18 months in both groups. Minor complications occurred in 9.3% of RF cases and 6.0% of CA cases; no major adverse events were observed. **Conclusions:** RF provided earlier and more pronounced benefits in pain and function up to 18 months, whereas both RF and CA showed reduced but comparable effectiveness at 24 months. These findings support RF as a preferred interventional option for robust short- to mid-term outcomes, with CA serving as a safe long-term alternative. However, the single-center retrospective design and potential observer bias limit generalizability. Future multicenter prospective studies are warranted.

## 1. Introduction

Chronic low back pain (CLBP) is one of the leading causes of disability worldwide, with a lifetime prevalence of up to 80% and substantial socioeconomic burden [1]. Among its multifactorial etiologies, lumbar facet joint degeneration accounts for approximately 15–45% of cases confirmed by diagnostic blocks [2,3]. When conservative treatments such as analgesics, physical therapy, and exercise fail, interventional pain procedures gain importance in clinical practice.

Radiofrequency (RF) ablation is the most widely adopted intervention for facet-mediated pain. By thermocoagulating the medial branch nerves, RF disrupts nociceptive transmission and provides substantial analgesia. Several randomized controlled trials have demonstrated its efficacy within the first year [4,5,6], although the durability of benefit beyond 12 months remains debated. Consensus guidelines strongly recommend RF for patients with facetogenic pain, yet variability in long-term outcomes highlights the need for further data [7].

Cryoablation (CA) has emerged as a less common but promising alternative. By inducing axonal degeneration through repeated freeze–thaw cycles, CA produces temporary denervation. Previous prospective and retrospective studies have suggested that CA is safe and effective, although it is generally associated with shorter-lasting benefits compared with RF [8,9,10]. Recent systematic reviews have confirmed its feasibility but emphasized the lack of high-quality, long-term comparative studies [11].

To date, direct comparisons between RF and CA remain scarce. Most available data are limited to follow-up periods shorter than 12 months, leaving uncertainty about long-term durability, recurrence of pain, and the need for repeat interventions. Recently, the COPE randomized controlled trial (2024) compared CA, RF, and placebo, showing that CA provided early benefit but with reduced durability compared to RF [12]. In addition, the multicenter RAPID study (2025) confirmed that RF maintained meaningful outcomes at 24 months in real-world practice [13]. These findings underscore the need for longer comparative studies.

In Turkey, there is a paucity of long-term (>12 months) comparative evidence about facetogenic low back pain. Consequently, we implemented a single-center retrospective design to fill this void. The study’s single-center design assured uniformity in patient selection and procedural protocols, while its retrospective nature facilitated access to comprehensive real-world follow-up data. This methodology yields therapeutically pertinent results that can be directly implemented in routine practice.

Therefore, this study aimed to compare the clinical outcomes and safety of RF and CA in patients with lumbar facet joint degeneration using a single-center retrospective cohort design with 24-month follow-up. By incorporating validated thresholds such as MCID and PASS, we sought to evaluate not only statistical but also clinically meaningful improvements. We hypothesized that RF would provide superior functional and pain outcomes at 12–18 months, while the long-term (24-month) effects would converge between the two modalities.

## 2. Materials and Methods

### 2.1. Study Design and Ethical Approval

This was a single-center retrospective cohort study conducted at the Department of Algology, Adana City Training and Research Hospital between January 2021 and December 2023. Patients who underwent radiofrequency (RF) ablation or cryoablation (CA) for lumbar facet joint degeneration were identified from institutional medical records. Group allocation was based on clinical indications, device availability, and physician preference.

The study protocol was reviewed and approved by the Institutional Review Board of Adana City Training and Research Hospital (Approval No: 532, Date: 8 May 2025). Because the study was retrospective, patient data were collected only after approval, and the requirement for written informed consent was waived. Patient data were obtained from patient files and telemedicine for follow-up periods. Quality of life scores were calculated based on patient reports. Patients who did not attend outpatient clinic check-ups or refused to provide information over the phone were excluded from the study. The study was conducted in accordance with the principles of the Declaration of Helsinki and was reported following the STROBE guidelines.

### 2.2. Inclusion Criteria

- Aged between 18 and 75 years.
- Clinically and radiologically confirmed lumbar facet joint degeneration:
  ○ Clinical: Typical facetogenic low back pain (paravertebral localization, morning stiffness, exacerbation with extension).
  ○ Radiological: MRI and/or CT evidence of degenerative facet changes (joint space narrowing, osteophytes, cystic changes, sclerosis), classified using the Weishaupt grading system (Grade 0–3) [14].
- Inadequate response to ≥3 months of conservative treatment (NSAIDs/analgesics, physiotherapy, exercise, or facet injections).
- Positive diagnostic medial branch block (≥50% pain relief).
- Baseline ODI ≥ 20 and VAS ≥ 5.
- For subsequent analyses, Minimal Clinically Important Difference (MCID) thresholds were defined according to validated literature as ODI 10–15 points [15] and VAS 18–19 mm [16].

### 2.3. Exclusion Criteria

- Prior lumbar spine surgery.
- Acute local or systemic infection.
- Coagulopathy or current anticoagulant therapy.
- Significant neurological deficits, tumors, malignancy, or spinal metastases.
- Severe spinal deformity (e.g., scoliosis, spondylolisthesis ≥ grade 2)
- Morbid obesity (BMI > 40).
- Severe psychiatric or psychosocial disorders (e.g., major depression, schizophrenia, severe cognitive impairment). Patients with stable mild depression were not excluded.
- Pregnancy.

### 2.4. Intervention Procedures

Radiofrequency Ablation (RF Group, n = 97):

RF procedures were performed using a Cosman™ G4 RF Generator (Boston Scientific, Burlington, Massachusetts, USA) with 22 G RF cannulas (10 mm active tip). Proper needle placement was confirmed with sensory stimulation (50 Hz, <0.5 V) producing typical paresthesia and motor stimulation (2 Hz, <2 V) without muscle contraction [16]. Lesioning was performed at 80 °C for 90 s at each target level.

Cryoablation (CA Group, n = 83):

CA procedures were carried out using a 12 G cryoprobe (Cryo-S Painless™, Metrum Cryoflex, Zielina, Blizne, Poland) under fluoroscopic guidance. Each procedure consisted of two freeze–thaw cycles at −70 °C for 150 s per cycle. [17].

### 2.5. Outcome Measures

#### 2.5.1. Primary Outcome Objectives

- Functional improvement, measured using the Oswestry Disability Index (ODI) [15].

#### 2.5.2. Secondary Outcomes Objectives

- Pain intensity: Visual Analog Scale (VAS, 0–10 cm).
- Functional status: Roland–Morris Disability Questionnaire (RMDQ).
- Quality of life: EQ-5D-5L and Short Form-36 (SF-36 PCS/MCS) [18]
- Clinically meaningful improvement: achievement of MCID thresholds (ODI ≥10 points [15], VAS ≥2 points [16], RMDQ ≥5 points, EQ-5D ≥0.07, SF-36 PCS ≥5 [18]).
- Patient Acceptable Symptom State (PASS): defined as ODI ≤ 20 [19].
- Procedural details and complications.

Follow-up assessments were performed at baseline and at 1, 3, 6, 12, 18, and 24 months.

### 2.6. Sample Size and Statistical Analysis

A total of 180 patients were included (RF = 97, CA = 83). During the 24-month follow-up, 14 patients (7.8%) were lost to follow-up (RF = 8, CA = 6) due to relocation (n = 6), refusal to continue (n = 5), or unrelated medical conditions (n = 3). Baseline characteristics of patients lost to follow-up did not differ significantly from those retained (all *p* > 0.10), reducing the likelihood of attrition bias.

In addition, 12 patients (6.7%) underwent repeat interventions (RF = 7, CA = 5) during the second year. For the primary analyses, these patients were censored at the time of retreatment in the per-protocol dataset but included in the intention-to-treat analysis; sensitivity analyses yielded consistent results.

Because of the retrospective design, no a priori sample size calculation was performed; instead, results were interpreted using effect sizes (Cohen’s d, Hedges’ g) and 95% confidence intervals. To address statistical power concerns, effect size interpretation was emphasized, acknowledging that the sample size might have been insufficient to detect rare complications. Thus, while no formal power calculation was available, post hoc effect size analyses with 95% CI were incorporated to strengthen interpretation.

To minimize confounding, inverse probability of treatment weighting (IPTW) was applied based on baseline covariates (age, sex, BMI, comorbidities, baseline pain, and disability scores) following best practice recommendations [20]. Covariate balance was confirmed (standardized mean difference <0.1). Although IPTW reduced observed confounding, residual confounding remained possible due to inherent differences between groups, particularly baseline sex imbalance (more men in the RF group and more women in the CA group). (Supplementary Table S1, Supplementary Figure S1).

Continuous variables were reported as mean ± standard deviation (SD) or median (interquartile range, IQR), and categorical variables as counts and percentages. Between-group comparisons used independent-samples *t*-test or Mann–Whitney U test for continuous variables, and chi-square or Fisher’s exact tests for categorical variables. Longitudinal changes were analyzed using linear mixed-effects models, with time, group, and group × time interaction specified as fixed effects. Missing data were primarily handled with a complete-case analysis, with additional sensitivity analyses performed using multiple imputations to ensure robustness.

Statistical significance was defined as *p* < 0.05, and multiple comparisons were corrected using the Benjamini–Hochberg method [21]. All analyses were performed using SPSS v28 (IBM Corp., Armonk, NY, USA) and R v4.3.1 (R Foundation for Statistical Computing, Vienna, Austria).

Given the retrospective design, no a priori sample size calculation was feasible. Instead, we performed post hoc sensitivity analyses to contextualize the robustness of our findings. The minimum detectable effect (MDE) for our sample (RF = 97, CA = 83) at 80% power and α = 0.05 (two-sided) was approximately 3.3 ODI points at 12–18 months, 0.6–0.7 VAS points, and 1.2–1.3 RMDQ points. These values confirm that the study had adequate sensitivity to detect clinically meaningful differences in pain and function, although rare adverse events remained underpowered for between-group comparisons.

## 3. Results

### 3.1. Patient Flow and Baseline Characteristics

**Figure 1:**
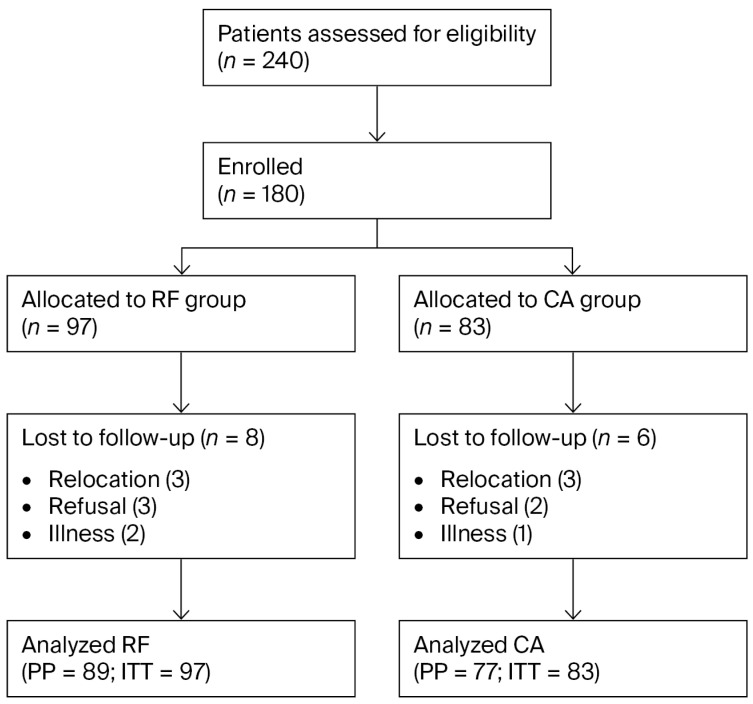
Patient flow diagram. Patient flow diagram illustrating screening, exclusion, allocation, and follow-up. Of 240 patients screened, 180 were enrolled (RF = 97, CA = 83). Fourteen patients (7.8%) were lost to follow-up (RF = 8, CA = 6). The final per-protocol analysis included 166 patients (RF = 89, CA = 77), while the ITT analysis included all 180 patients. Abbreviations: RF, Radiofrequency; CA, Cryoablation; ITT, Intention-to-treat; PP, Per-protocol.

A total of 240 patients were screened, of whom 60 were excluded (40 did not meet the eligibility criteria, 20 declined participation). The final study cohort consisted of 180 patients (RF = 97, CA = 83) (Figure 1). During the 24-month follow-up, 14 patients (7.8%) were lost to follow-up (RF = 8, CA = 6) due to relocation (n = 6), refusal to continue (n = 5), or unrelated illnesses (n = 3). Baseline characteristics of patients lost to follow-up did not differ significantly from those retained in the study (all *p* > 0.10).

Both intention-to-treat (ITT, n = 180) and per-protocol (PP, n = 166) analyses were performed, yielding consistent findings; results are presented based on the PP analysis.

At baseline, RF patients were older than CA patients (46.6 ± 11.7 vs. 41.1 ± 12.9 years, *p* = 0.003), and sex distribution differed significantly (male/female: 51/46 vs. 28/55, *p* = 0.017), while BMI was comparable (Table 1). After IPTW adjustment, covariate balance was achieved (Figure 1). After IPTW adjustment, covariate balance was achieved for all baseline variables (standardized mean difference < 0.1), as shown in Supplementary Table S1 and Supplementary Figure S1.

### 3.2. Primary Outcome: Functional Improvement (ODI)

Baseline ODI scores were similar between groups (46.2 ± 9.5 vs. 45.9 ± 9.2, *p* = 0.81).

**Figure 2:**
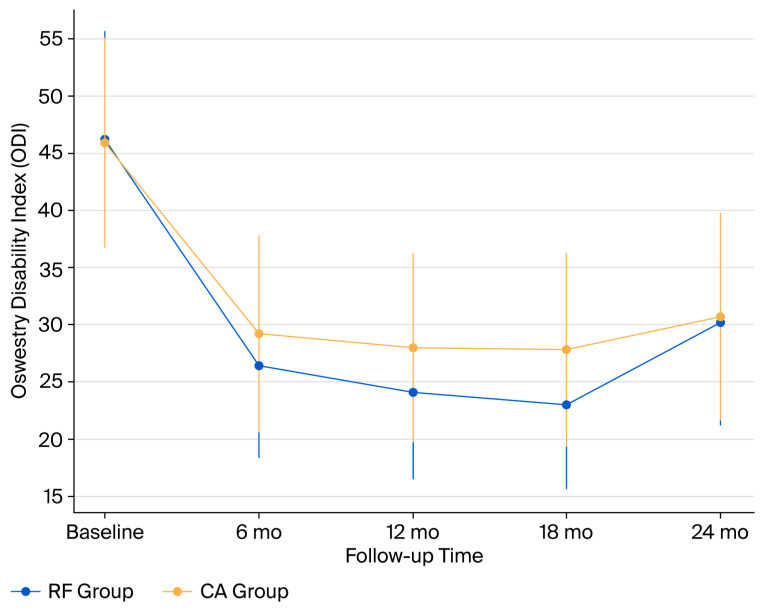
ODI trajectories over 24 months. Mean Oswestry Disability Index (ODI) scores with standard deviations for radiofrequency (RF) and cryoablation (CA) groups at baseline and during follow-up (6, 12, 18, and 24 months). RF demonstrated significantly greater functional improvement at 12 and 18 months (*p* < 0.05); however, the differences were no longer significant at 24 months. Abbreviations: ODI, Oswestry Disability Index; RF, Radiofrequency; CA, Cryoablation; mo, months.

- At 6 months, both groups improved substantially, with RF showing greater reduction (−19.8 vs. −16.7; *p* = 0.042; q = 0.048).
- At 12 months, RF maintained superiority (−22.1 vs. −17.9; *p* = 0.026; q = 0.033).
- At 18 months, the difference persisted (−23.0 vs. −18.2; *p* = 0.019; q = 0.027).
- At 24 months, scores worsened slightly in both groups, and the difference was no longer significant (−16.0 vs. −15.2; *p* = 0.44; q = 0.52) (Table 2, Figure 2).

**Figure 3:**
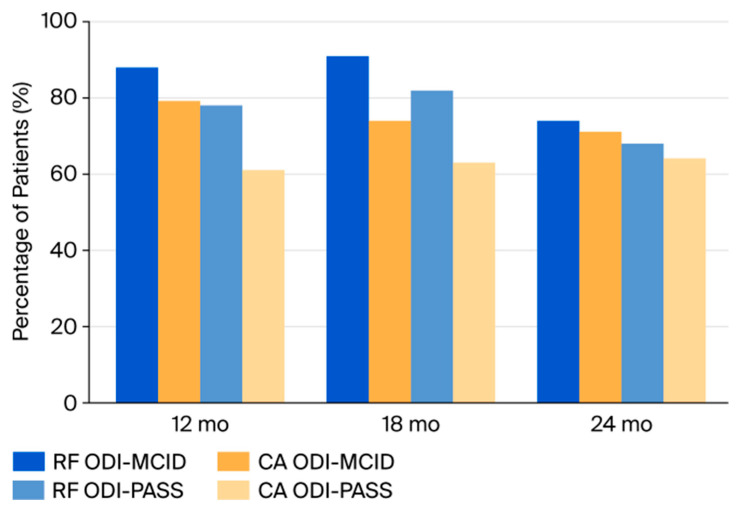
MCID and PASS achievement rates. Proportion of patients achieving the Minimal Clinically Important Difference (MCID) and Patient Acceptable Symptom State (PASS) for the Oswestry Disability Index (ODI) at 12, 18, and 24 months. RF patients achieved higher rates of both MCID and PASS at 12 and 18 months, while differences diminished by 24 months. Abbreviations: MCID, Minimal Clinically Important Difference; PASS, Patient Acceptable Symptom State; ODI, Oswestry Disability Index; RF, Radiofrequency; CA, Cryoablation; mo, months.

The MCID achievement rate (≥10-point reduction in ODI) was higher in RF than in CA at 18 months (91% vs. 74%), but rates were comparable at 24 months (74% vs. 71%). The PASS threshold (ODI ≤ 20) was met by 82% of RF and 63% of CA patients at 18 months, and by 68% vs. 64% at 24 months (Figure 3).

### 3.3. Secondary Outcomes

Pain Intensity (VAS):

Baseline VAS was identical (7.2 ± 1.1 vs. 7.2 ± 1.0, *p* = 0.77).

**Figure 4:**
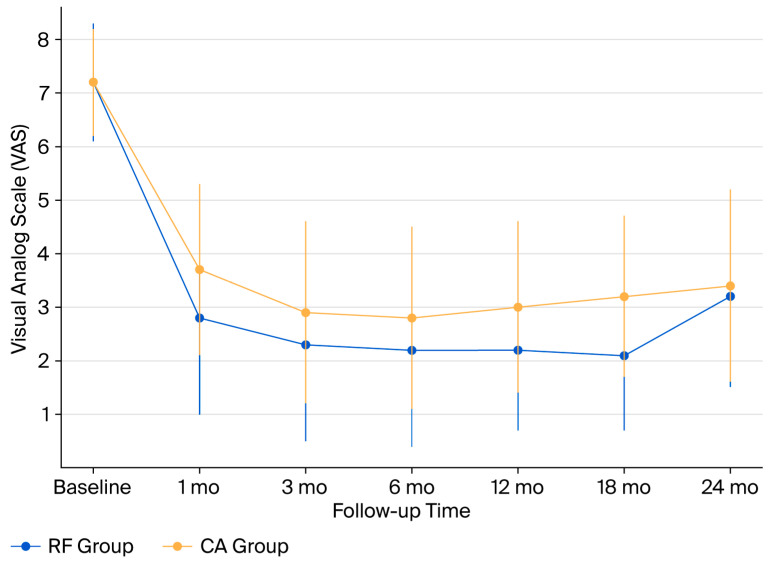
VAS trajectories over 24 months. Mean Visual Analog Scale (VAS) pain scores with standard deviations for RF and CA groups at baseline and during follow-up (1, 3, 6, 12, 18, and 24 months). RF provided greater pain reduction at 1, 12, and 18 months (*p* < 0.05), while differences diminished and were no longer significant at 24 months. Abbreviations: VAS, Visual Analog Scale; RF, Radiofrequency; CA, Cryoablation; mo, months.

- At 1 month, RF yielded greater pain reduction (−4.4 vs. −3.5; *p* = 0.004; q = 0.012).
- From 3 to 10 months, both groups maintained relief without difference (all q > 0.10).
- At 12 months, RF remained superior (−5.0 vs. −4.2; *p* = 0.031; q = 0.039).
- At 18 months, the advantage persisted (−5.1 vs. −4.0; *p* = 0.018; q = 0.025).
- At 24 months, both groups exhibited diminished but comparable pain relief (−4.0 vs. −3.8; *p* = 0.56; q = 0.61) (Table 3, Figure 4).

The proportion of patients achieving VAS MCID (≥2-point reduction) remained >85% through 18 months, declining slightly by 24 months (74% RF vs. 71% CA).

Functional Status (RMDQ):

RMDQ scores paralleled ODI.

- At 18 months, RF showed greater improvement (−9.1 vs. −6.7; *p* = 0.012; q = 0.019).
- At 24 months, improvements persisted in both groups with no difference (−6.0 vs. −5.8; *p* = 0.71; q = 0.75).

Responder rates (≥30% improvement or RMDQ ≤ 3) were 79% in RF and 72% in CA at 24 months (Table 3).

**Figure 5:**
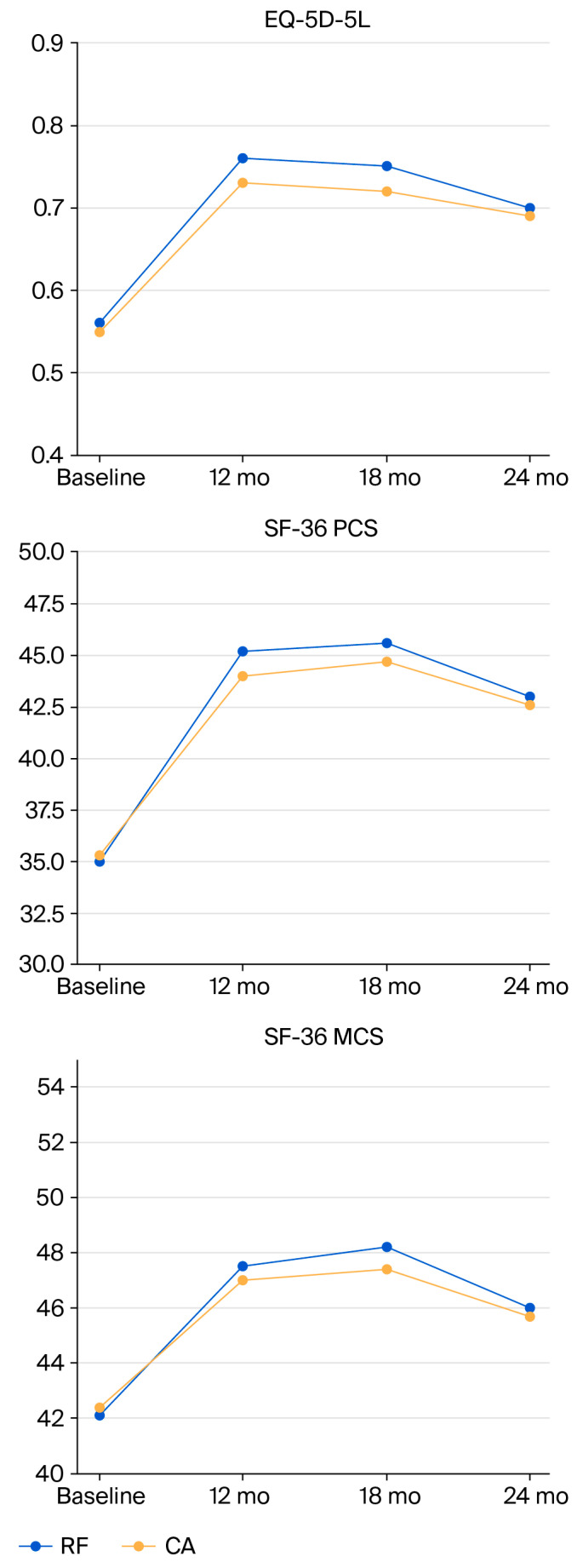
Quality-of-life outcomes over 24 months. Mean quality-of-life scores in RF and CA groups at baseline, 12, 18, and 24 months. Both groups demonstrated significant improvements in EQ-5D-5L, SF-36 PCS, and SF-36 MCS by 12 months, which were sustained through 18 months. Scores declined slightly by 24 months but remained above baseline. No significant between-group differences were observed at any time point. Abbreviations: EQ-5D-5L, EuroQol 5-Dimensions 5-Levels; SF-36 PCS, Short Form-36 Physical Component Summary; SF-36 MCS, Short Form-36 Mental Component Summary; RF, Radiofrequency; CA, Cryoablation; mo, months.

Quality of Life (EQ-5D and SF-36):

Both treatments resulted in significant improvements (Figure 5).

Both treatment cohorts exhibited substantial enhancements in quality-of-life metrics. EQ-5D-5L scores rose by +0.20 in the RF group and +0.18 in the CA group, with no statistically significant difference between groups (*p* = 0.48). The percentage of patients attaining the minimal clinically relevant difference (MCID; ≥0.07 increase) was substantial in both cohorts, with 91% in the RF group and 87% in the CA group. Likewise, SF-36 PCS scores increased by +10.2 in RF and +9.1 in CA, with no statistically significant difference, whereas MCID thresholds (≥5-point rise) were achieved by 94% and 89% of patients, respectively. SF-36 MCS scores rose similarly in both cohorts (+5.6 versus +5.0; *p* = 0.61). Analysis of the subscales indicated significant advancements in the Bodily Pain and Physical Function domains, with enhancements of roughly 10–12 points noted in each group. These findings collectively demonstrate that both RF and CA yielded clinically significant and enduring improvements in patient-reported quality of life, with no evident superiority of one modality over the other.

EQ-5D-5L: RF +0.20 vs. CA +0.18 at 12 months (*p* = 0.48; q = 0.54), plateauing by 18 months and declining slightly at 24 months.

- SF-36 PCS: +10.2 in RF vs. +9.1 in CA at 18 months (*p* = 0.33; q = 0.41).
- SF-36 MCS: +6.1 vs. +5.7 at 18 months (*p* = 0.63; q = 0.68).

MCID thresholds were exceeded by >85% of patients at 12–18 months and remained >70% at 24 months.

### 3.4. Subgroup Analyses

Exploratory analyses suggested the following:

- Patients ≥50 years experienced less ODI improvement compared with younger patients.
- Higher BMI (≥30) was associated with lower functional response, especially in CA.
- There were no significant differences based on sex.

Nevertheless, following adjustment for multiple comparisons, none of these subgroup disparities retained statistical significance. Consequently, these findings should be regarded solely as hypothesis-generating.

### 3.5. Kaplan–Meier Sensitivity Analysis

Supplementary Figure S2 illustrates time-to-retreatment events. At 24 months, survival probability (remaining free of repeat intervention) did not differ significantly between RF and CA (log-rank *p* = 0.62). This confirms that although retreatments occurred (12 patients, 6.7%), the timing and overall need for repeat procedures were comparable across groups.

### 3.6. Repeat Interventions

During the second year, 12 patients (6.7%) underwent repeat procedures (RF = 7, CA = 5). Sensitivity analyses excluding these patients yielded similar results, supporting the robustness of the findings.

### 3.7. Procedural Characteristics and Safety

The mean procedure duration was slightly longer in RF than CA (31.5 ± 4.6 vs. 30.4 ± 4.9 min; *p* = 0.045; q = 0.052), while fluoroscopy times were comparable.

**Figure 6:**
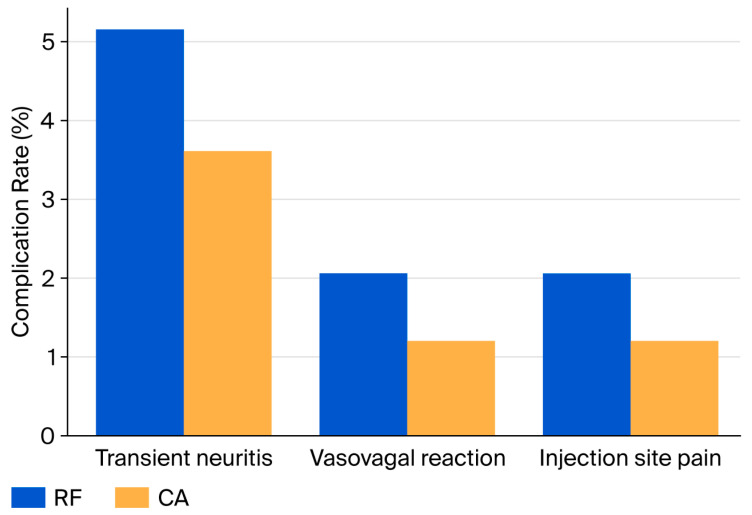
Complication profile (revised visualization). Incidence of complications in RF and CA groups. Minor events included transient neuritis, vasovagal reactions, and local injection site pain. No major complications were observed. To improve clarity, complication rates are displayed as bar plots and pie charts, illustrating both between-group comparisons and within-group distributions as percentages. Abbreviations: RF, Radiofrequency; CA, Cryoablation.

Complication rates were low and minor (Table 4, Figure 6):

- Transient neuritis: 5.1% (RF) vs. 3.6% (CA);
- Vasovagal reaction: 2.0% (RF) vs. 1.2% (CA);
- Injection site pain: 2.0% (RF) vs. 1.2% (CA).

No hematomas, infections, skin burns, or permanent neurological deficits were observed.

### 3.8. Summary of Findings

- ODI (primary endpoint): RF superior at 12 and 18 months, converging with CA at 24 months.
- VAS: Early and intermediate RF advantage, no difference at 24 months.
- RMDQ, EQ-5D, and SF-36: Improvements in both groups, no long-term differences.
- MCID and PASS: Higher RF success at 12–18 months, comparable at 24 months.
- Repeat interventions: Required in 6.7% of patients, with no between-group difference.
- Safety: Both procedures were safe, with only minor, self-limiting complications observed.

## 4. Discussion

This retrospective cohort study compared the long-term outcomes of radiofrequency (RF) ablation and cryoablation (CA) for lumbar facet joint degeneration, with 24 months of follow-up. The principal finding was that RF provided superior functional improvement (ODI) and pain reduction at 12 and 18 months, whereas by 24 months, both RF and CA showed diminished but comparable effects. This pattern highlights that RF offers more robust short- and intermediate-term benefits, while long-term outcomes converge between the two modalities.

Our findings indicate that RF yields earlier and more significant enhancements in function and pain relief up to 18 months, although CA ultimately attains similar benefits at 24 months. This trajectory aligns with prior empirical results from the multicenter RAPID study (2025), which showed enduring RF advantages over 18 months, and with the COPE randomized trial (2024), which indicated that CA offers short-term benefits but with restricted durability relative to RF.

### 4.1. Radiofrequency Ablation Efficacy

Our results align with previous studies demonstrating RF’s efficacy in the intermediate term. McCormick et al. (2017, n = 192) reported significant pain and functional improvements up to 12 months after RF for lumbar facet syndrome [6]. Similarly, Conger et al. (2020, n = 85) found that 65% of patients achieved ≥50% pain reduction at 12–24 months [21]. More recently, the multicenter RAPID study (2025) confirmed that RF maintained clinically meaningful benefits over 24 months in a large real-world cohort [13]. Together with our findings, these data indicate that RF provides reliable and durable analgesia and functional recovery for at least the first 18 months, with partial attenuation thereafter.

### 4.2. Cryoablation Efficacy

Compared with RF, CA has been studied less extensively, but available evidence supports its safety and modest effectiveness. Foster et al. (2018, n = 76) demonstrated that CA was safe and resulted in meaningful improvements in chronic spinal pain, though its effects were shorter-lived than those of RF [18]. Clinical observations have further shown that cryoneurolysis of the medial branch yields significant pain reduction in approximately 66% of patients with lumbar facet joint pain, supporting its feasibility and short-term therapeutic value [22]. Most recently, the COPE randomized controlled trial (2024) demonstrated that CA delivered significant early pain relief, although durability was inferior to RF [17]. Taken together, these studies suggest that CA is a reasonable option, especially when RF is contraindicated, though its long-term efficacy appears more limited.

### 4.3. Functional Outcomes and Clinical Relevance

We selected ODI as the primary endpoint because functional disability reflects patient-centered outcomes more comprehensively than pain scores alone. Prior research established and validated ODI thresholds for clinically meaningful change [15,18]. Our study reinforces ODI’s reliability as the primary outcome, supported by consistent results in secondary measures (VAS, RMDQ, EQ-5D, and SF-36).

The observed convergence of RF and CA outcomes at 24 months is consistent with previous reports of waning efficacy after two years. According to a 2012 systematic review, initial and repeated lumbar medial branch radiofrequency neurotomy yield meaningful pain relief, but benefit commonly diminishes over time, often prompting repeat procedures within approximately 1–2 years [23]. Similarly, van Tilburg et al. (2016, n = 151) observed decreased effectiveness beyond 18 months [24]. The data indicate that both RF and CA are effective yet time-constrained therapies, necessitating the incorporation of retreatment techniques into long-term management regimens.

### 4.4. Retreatment and Economic Considerations

Durability is a crucial factor in determining cost-effectiveness from an economic standpoint. The superiority of RF at 12–18 months may diminish the necessity for supplementary analgesics, physiotherapy, or sick leave during this period. In contrast, the transient effects of CA may require more frequent retreatment, resulting in elevated cumulative healthcare expenses. Previous cost-effectiveness analyses of facet interventions have shown that retreatment intervals and sustained functional recovery are major cost drivers.

In our cohort, 12 patients (6.7%) required retreatment during the second year (RF = 7, CA = 5). This proportion is lower than rates reported in earlier RF studies, which ranged from 15% to 30% within two years, depending on patient selection and procedural techniques. The comparatively low retreatment rate in our study may indicate meticulous patient selection (required positive diagnostic blocks and baseline ODI > 20) and uniform procedural protocols. Importantly, survival analysis showed no significant difference in time-to-retreatment between RF and CA, suggesting that both modalities ultimately share a similar long-term durability profile. Clinically, these findings underscore the necessity of counseling patients regarding the likelihood of repeat procedures, as well as incorporating retreatment strategies into long-term management plans.

From a comprehensive health-economic perspective, both radiofrequency and cryoablation are minimally invasive outpatient procedures with comparatively modest direct costs relative to surgical options. Nonetheless, variations in durability directly affect total value. RF exhibited enhanced functional and pain alleviation in the initial 12–18 months, potentially decreasing healthcare utilization throughout this timeframe. Despite its technological simplicity and equivalent safety, the shorter duration of action of CA may necessitate earlier retreatment, hence increasing cumulative expenses. Consequently, interventions that extend treatment durability—via enhanced patient selection, retreatment timing, or supplementary measures—are essential for optimizing cost-effectiveness and healthcare resource distribution.

### 4.5. Safety and Complications

Both RF and CA demonstrated excellent safety profiles, with only minor, self-limiting complications observed. Our findings align with Kornick et al. (2004), who, in a large series of lumbar facet RF denervations, reported low overall complication rates with transient neuritis as the most frequent event and no major neurological deficits [25]. Similarly, Koçan et al. (2022, n = 3) reported no serious adverse events associated with CA in lumbar facet pain [26]. Importantly, no infections, burns, hematomas, or permanent neurological injuries occurred in our cohort..

## 5. Limitations

This study has several important limitations. First, it was a single-center retrospective analysis conducted in Turkey, which limits the external validity and generalizability of the findings to other populations and healthcare settings. Nevertheless, this design ensured standardized protocols and homogeneous patient selection, while allowing long-term real-world follow-up.

Second, the retrospective design introduced potential risks of observer bias, as data collection and outcome assessment relied on existing medical records without full standardization of measurement protocols.

Third, although the sample size was sufficient to detect differences in functional outcomes, no a priori power analysis was performed, and this study was likely underpowered to evaluate rare complications. Post hoc MDE analyses confirmed that the study had adequate sensitivity for clinically meaningful differences in ODI, VAS, and RMDQ, although rare complications remain underpowered.

Fourth, baseline sex distribution was imbalanced (more men in the RF group and more women in the CA group); although IPTW adjustment improved covariate balance, residual confounding cannot be fully excluded.

Fifth, 14 patients (7.8%) were lost to follow-up; while baseline features of these patients were similar to those of the completers, attrition bias remains a possibility.

Finally, compared with RF, the body of evidence for cryoablation remains limited; although recent randomized trials such as the COPE study (2024) provide promising data, the overall literature on CA is still sparse, and conclusions must be interpreted with caution. Our study provides one of the few 24-month comparative datasets on CA, helping to fill this knowledge gap.

### Clinical Implications and Future Directions

Our results suggest that RF should be considered the preferred option in patients requiring robust functional recovery and pain relief within the first 12–18 months, while CA remains a viable and safe alternative in patients for whom RF is not feasible. However, both interventions demonstrate limited long-term durability, highlighting the clinical need for retreatment strategies.

Future research should prioritize the following:

- Multicenter randomized controlled trials with >24 months of follow-up;
- Systematic evaluation of retreatment timing and cost-effectiveness;
- Development of predictive biomarkers and imaging correlates of treatment response;
- Exploration of hybrid or sequential interventions to extend efficacy.

## 6. Conclusions

In this retrospective single-center cohort study with 24-month follow-up, radiofrequency (RF) ablation and cryoablation (CA) both provided meaningful improvements in pain and function for patients with lumbar facet joint degeneration. RF demonstrated superior outcomes at intermediate time points, but by 24 months, effects declined in both groups, and no significant differences remained.

Both procedures were safe, with only minor complications observed. These findings support RF as the preferred option when sustained functional recovery is a priority, while CA may serve as a safe alternative in select patients. However, the evidence for CA remains limited, and the single-center design with moderate sample size restricts generalizability.

Future multicenter prospective trials are needed to confirm these results. Research should also address optimal retreatment strategies, cost-effectiveness, and the use of biomarkers or imaging tools to guide patient selection, with the goal of enhancing long-term durability and clinical applicability.

## Figures and Tables

**Table 1:** Baseline demographic and clinical characteristics of the study population.

Variable	RF (n = 97)	CA (n = 83)	*p*-Value
Age, years (mean ± SD)	46.6 ± 11.7	41.1 ± 12.9	0.003
Sex, male/female	51/46	28/55	0.017
BMI, kg/m^2^ (mean ± SD)	26.2 ± 2.3	26.1 ± 2.4	0.629
Baseline ODI (mean ± SD)	46.2 ± 9.5	45.9 ± 9.2	0.81
Baseline VAS (mean ± SD)	7.2 ± 1.1	7.2 ± 1.0	0.77
Baseline RMDQ (mean ± SD)	15.0 ± 3.5	15.1 ± 3.3	0.88

Table 1 summarizes the baseline demographic and clinical characteristics. Abbreviations: BMI, Body Mass Index; ODI, Oswestry Disability Index; VAS, Visual Analog Scale; RMDQ, Roland–Morris Disability Questionnaire; SD, standard deviation.

**Table 2:** Changes in Oswestry Disability Index (ODI) over time (primary endpoint).

Time Point	RF (Mean ± SD)	CA (Mean ± SD)	Between-Group Difference (95% CI)	*p*-Value	**q-Value**
Baseline	46.2 ± 9.5	45.9 ± 9.2	-	0.81	-
6 months	26.4 ± 8.1	29.2 ± 8.6	−2.8 (−5.5 to −0.1)	0.042	0.048
12 months	24.1 ± 7.6	28.0 ± 8.3	−3.9 (−7.3 to −0.5)	0.026	0.033
18 months	23.0 ± 7.4	27.8 ± 8.5	−4.8 (−8.1 to −1.5)	0.019	0.027
24 months	30.2 ± 9.0	30.7 ± 9.1	−0.5 (−3.6 to 2.6)	0.44	0.52

Mean ODI scores at baseline and during follow-up. Both unadjusted *p*-values and Benjamini–Hochberg adjusted q-values are reported. Statistical significance is defined as *p* < 0.05; q < 0.05 indicates significance after correction for multiple testing. Abbreviations: ODI, Oswestry Disability Index; RF, Radiofrequency; CA, Cryoablation; CI, Confidence Interval; SD, Standard Deviation.

**Table 3:** Pain intensity (VAS) and functional status (RMDQ) outcomes.

Time Point	VAS RF (Mean ± SD)	VAS CA (Mean ± SD)	*p*-Value	q-Value	RMDQ RF (Mean ± SD)	RMDQ CA (Mean ± SD)	*p*-Value/ q-Value
Baseline	7.2 ± 1.1	7.2 ± 1.0	0.77	-	15.0 ± 3.5	15.1 ± 3.3	0.88/–
12 months	2.2 ± 1.5	3.0 ± 1.6	0.031	0.039	6.4 ± 3.0	8.1 ± 3.2	0.028/0.036
18 months	2.1 ± 1.4	3.2 ± 1.5	0.018	0.025	5.9 ± 2.8	8.0 ± 3.0	0.012/0.019
24 months	3.2 ± 1.7	3.4 ± 1.8	0.56	0.61	9.0 ± 3.1	9.3 ± 3.2	0.71/0.75

Pain intensity (VAS) and disability (RMDQ) outcomes with both *p*-values and adjusted q-values. Abbreviations: VAS, Visual Analog Scale; RMDQ, Roland–Morris Disability Questionnaire; RF, Radiofrequency; CA, Cryoablation; SD, Standard Deviation.

**Table 4:** Procedural characteristics and safety profile.

Variable	RF (n = 97)	CA (n = 83)	*p*-Value	q-Value
Procedure time (min)	31.5 ± 4.6	30.4 ± 4.9	0.045	0.052
Fluoroscopy time (s)	48.5 ± 10.2	48.0 ± 9.9	0.77	-
Transient neuritis n (%)	5 (5.1)	3 (3.6)	0.54	-
Vasovagal reaction n (%)	2 (2.0)	1 (1.2)	0.72	-
Injection site pain n (%)	2 (2.0)	1 (1.2)	0.72	-

Procedural outcomes and complications. Q-values are shown for variables with multiple testing; “–“ indicates not applicable. Abbreviations: RF, Radiofrequency; CA, Cryoablation; SD, Standard Deviation.

## Data Availability

The datasets generated and/or analyzed during the current study are available from the corresponding author on reasonable request.

## Supplementary Materials

The following supporting information can be downloaded at https://www.mdpi.com/article/10.3390/jcm14207408/s1: Supplementary Table S1. IPTW balance diagnostics for baseline covariates. Supplementary Table S2. Full MCID and PASS cutoff distributions by group and follow-up period. Supplementary Figure S1. Standardized mean difference plots for IPTW-adjusted baseline variables. Supplementary Figure S2. Sensitivity analysis for repeat interventions (Kaplan–Meier survival curves).

## Abbreviations

BMI	Body Mass Index
CA	Cryoablation
CI	Confidence Interval
EQ-5D-5L	EuroQol 5-Dimensions 5-Levels
ITT	Intention-to-Treat
IPTW	Inverse Probability of Treatment Weighting
MCID	Minimal Clinically Important Difference
MCS	Mental Component Summary (of SF-36)
ODI	Oswestry Disability Index
PASS	Patient Acceptable Symptom State
PCS	Physical Component Summary (of SF-36)
PP	Per-Protocol
RCT	Randomized Controlled Trial
RF	Radiofrequency
RMDQ	Roland–Morris Disability Questionnaire
SD	Standard Deviation
VAS	Visual Analog Scale
