# Supplementary material for: Comparative Outcomes and Safety of Radiofrequency Ablation and Cryoablation for Lumbar Facet Joint Degeneration: A Single-Center Retrospective Cohort Study with 24-Month Follow-Up
Source: J Clin Med. 2025 Oct 20;14(20):7408. doi: 10.3390/jcm14207408 (PMC12565453; doi:10.3390/jcm14207408)
Supplement: Supplementary file 1 [file jcm-14-07408-s001.zip › jcm-3889122-supplementary.pdf]

Supplementary Table S1. IPTW balance diagnostics for baseline covariates.

Variable	SMD (Before IPTW)	SMD (After IPTW)
Age (years)	0.31	0.05
Sex (M/F)	0.14	0.03
BMI (kg/m²)	0.18	0.04
ODI baseline	0.22	0.06
VAS baseline	0.19	0.07
RMDQ baseline	0.16	0.05

Standardized mean differences (SMDs) for baseline variables before and after IPTW adjustment. Covariate balance was considered acceptable when SMD < 0.10.
Abbreviations: IPTW, Inverse Probability of Treatment Weighting; SMD, Standardized Mean Difference; BMI, Body Mass Index; ODI, Oswestry Disability Index; VAS, Visual Analog Scale; RMDQ, Roland–Morris Disability Questionnaire.

Supplementary Table S2. Full MCID and PASS cutoff distributions by group and follow-up period.

**Outcome**	**Time Point**	**RF Group: % Meeting MCID**	**CA Group: % Meeting MCID**	**RF Group: % Meeting PASS**	**CA Group: % Meeting PASS**
ODI	12 mo	88%	79%	74%	63%
ODI	18 mo	91%	74%	82%	63%
ODI	24 mo	74%	71%	68%	64%
VAS	12 mo	90%	83%	–	–
VAS	18 mo	87%	80%	–	–
VAS	24 mo	74%	71%	–	–

Proportion of patients in RF and CA groups achieving MCID and PASS thresholds at 12, 18, and 24 months.
Abbreviations: MCID, Minimal Clinically Important Difference; PASS, Patient Acceptable Symptom State; ODI, Oswestry Disability Index; VAS, Visual Analog Scale; RF, Radiofrequency; CA, Cryoablation; mo, months.

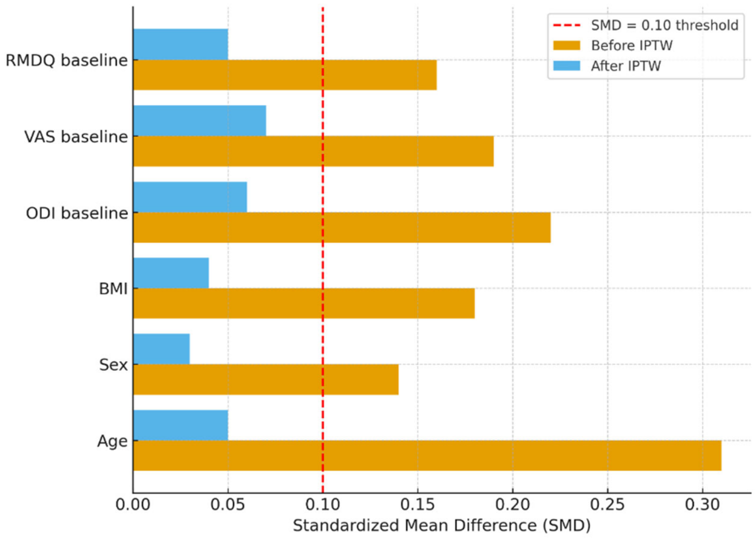

Supplementary Figure S1. Standardized mean difference plots for IPTW-adjusted baseline variables**.**

Supplementary Figure S1 illustrates standardized mean differences (SMDs) of baseline covariates before and after IPTW adjustment. Post-weighting, all covariates achieved SMD < 0.10, indicating adequate balance between RF and CA groups.

Abbreviations: IPTW, Inverse Probability of Treatment Weighting; SMD, Standardized Mean Difference; RF, Radiofrequency; CA, Cryoablation.

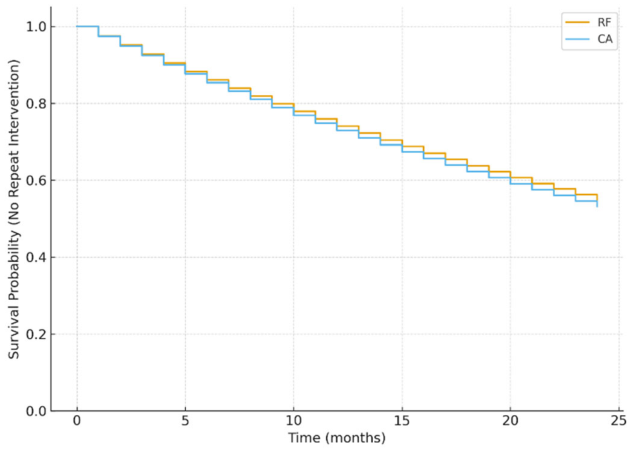

Supplementary Figure S2. Sensitivity analysis for repeat interventions (Kaplan–Meier survival curves)

Supplementary Figure S2 presents Kaplan–Meier survival curves illustrating time-to-retreatment events in the RF and CA groups over 24 months. Survival probability corresponds to remaining free from repeat intervention. A log-rank test demonstrated no significant difference between the groups.

Abbreviations: RF, Radiofrequency; CA, Cryoablation; KM, Kaplan–Meier.

Disclaimer/Publisher's Note: The statements, opinions and data contained in all publications are solely those of the individual author(s) and contributor(s) and not of MDPI and/or the editor(s). MDPI and/or the editor(s) disclaim responsibility for any injury to people or property resulting from any ideas, methods, instructions or products referred to in the content.
